# Regional variation in mineral content, cytotoxicity, and antioxidant activity of *Aerodramus fuciphagus* nests from different districts in Kelantan, Malaysia

**DOI:** 10.5455/javar.2025.l880

**Published:** 2025-03-24

**Authors:** Mohd Dasuki Sul’ain, Musa Isah, Wan-Nor-Amilah Wan Abdul Wahab

**Affiliations:** 1 School of Health Sciences, Universiti Sains Malaysia, Health Campus, Kota Bharu, Malaysia; 2 Department of Microbiology, Kebbi State University of Science and Technology Aliero, Nigeria

**Keywords:** *Aerodramus fuciphagus*, antioxidant activity, cytotoxicity, edible bird’s nest, mineral content

## Abstract

**Objective:**

This study aimed to assess *Aerodramus fuciphagus *(Thunberg, 1812) nest’s mineral content, cytotoxicity effects, and antioxidant activity. The Edible birds’ nests (EBNs) were obtained from Tanah Merah (TM), Tumpat (TU), and Jeli (JE) districts in Kelantan, Malaysia.

**Materials and Methods:**

The mineral content was assessed via inductively coupled plasma mass spectrometry. The cytotoxicity effect was evaluated by 3-[4,5-dimethylthiazol-2-yl]-2,5-diphenyl tetrazolium bromide and brine shrimp lethality assays. The antioxidant activity was investigated by 2,2´-diphenyl-2-picrylhydrazyl (DPPH) radical scavenging and ferric-reducing antioxidant power (FRAP) assays.

**Results:**

Valuable macro- and microelements, including sodium, potassium, aluminum, calcium, magnesium, zinc, iron, and selenium, were detected in the EBN samples. The cytotoxicity test revealed that JE hydrochloric acid (HCl) extract had promising efficacy against HeLa cell lines, with IC_50_ values of 20.00 ± 0.57 µg/ml. Similarly, TU sodium hydroxide (NaOH) extract inhibited the proliferation of MCF-7 cell lines (IC_50_ 0.03 ± 0.01 µg/ml). Based on the BSL assay, the EBNs were considered safe for consumption as the LC_50_ values of all the samples were below the toxic level (>1,000 µg/ml). TM EBNs aqueous, TU aqueous, and HCl extracts showed promising antioxidant activity at IC_50_ values (10 µg/ml) in the DPPH assay. However, the NaOH extract from TU exhibited the highest FRAP value (1.38 ± 0.00 mM gallic acid equivalents gm⁻¹) compared to all other extracts.

**Conclusion:**

This study’s findings demonstrated the potential of EBN as an alternative anticancer agent and natural antioxidant.

## Introduction

The curative properties of natural products have spurred a significant interest in the search for alternative remedies. Consequently, these natural products are considered a potential alternative to synthetic drugs, which are often associated with numerous adverse effects and prevailing multi-drug resistance [1,2]. Edible birds’ nests (EBNs) produced by swiftlet species have been traditionally used across Asian continent for their health-promoting properties. These species are widely distributed in Malaysia, Vietnam, Indonesia, and Thailand [3]. Asians have a long-standing tradition of consuming EBN, which has healing effects [4]. Several swiftlet species produce EBNs, such as the *Aerodramus* and *Collocalia* genera [5].

The *Aerodramus* genus consists of two main species: *Aerodramus fuciphagus* Thunberg 1812, often known as the white nest species, and *Aerodramus maximus* Hume 1878, the black nest species. The *A*.* fuciphagus* builds its nest with saliva produced by the swiftlet’s two sublingual glands. The *A*.* maximus* simultaneously makes its nest using saliva and feathers [6]. EBN products have been widely used for various purposes, including prestige, food supplements, and healthcare delivery; they are commonly used as a solution for malnutrition and a stimulant to boost the body’s metabolism [7].

Within the Chinese community, EBN is highly regarded for its exceptional qualities as a meal, medicinal remedy, and beauty enhancer [8,9]. The pharmacological effects of EBN can be ascribed to its enormous bioactive constituents, including fatty acids, minerals, vitamins, sialic acid, glucosamine, triacylglycerol, and amino acids [9,10]. Cancer is widely recognized as a highly distressing and burdensome illness, affecting millions of individuals globally each year [11]. Despite notable progress in therapeutics and diagnostics, the rise of drug resistance and tumor relapse has become increasingly prevalent [12]. Moreover, synthetic anticancer drugs have been associated with adverse effects, such as the possible emergence of uterine cancer, endometrial cancer, blood clots, cataracts, and stroke [13]. Therefore, exploring alternative drugs with greater efficacy from natural sources is necessary. Plants often serve as valuable reservoirs of natural antioxidants, which can efficiently neutralize reactive oxygen species [14]. Moreover, collagen, functional foods, and keratin are good sources of natural antioxidants [15]. Natural antioxidants are crucial in maintaining good health and regulating cellular metabolism [16].

Several studies have demonstrated that consuming a diet abundant in antioxidants can reduce the likelihood of developing chronic illnesses such as malignancies, heart ailments, and neurological disorders [17,18]. In addition, natural antioxidants enhance the immune system, foster healthy skin, and control inflammation [19]. According to [6,20,21], EBN predominantly consists of proteins, carbohydrates, and vital minerals such as calcium and sodium, with its composition influenced by geographical origin, nesting environment, and species. Furthermore, it is abundant in sialic acid and several bioactive components that augment antioxidant properties post-digestion, indicating its possible nutraceutical applications [4,15]. Even though EBN possesses biological activities such as anti-inflammatory, antioxidant, antiaging, and antimicrobial properties, there are knowledge gaps regarding its bioavailability, a lack of comparative studies across different origins, and insufficient data on its long-term safety and health effects [7,15,21]. Thus, this research aims to address critical knowledge gaps in EBN research by systematically analyzing the impact of regional variation on the nutrient composition, safety, and bioactivity of EBN extracts from different districts in Kelantan, Malaysia.

## Materials and Methods

### Sample collection

EBN samples were collected in May 2023 from the swiftlet house in the Tanah Merah (TM), Tumpat (TU), and Jeli (JE) districts in Kelantan, Malaysia. The Department of Veterinary Services Malaysia verified and confirmed the samples.

### Extract preparation

The extraction of EBN was carried out according to the method described by Lai et al. [10], involving initial cleaning with sterile distilled water and drying at 50°C for 12 h. The nest was then crushed into fine particles, sieved through a 0.4 mm screen, and suspended in solvents, including distilled water, sodium hydroxide (NaOH), and hydrochloric acid (HCl) under controlled conditions. The extracts were allowed to elute for 16 h at 4°C. A portion of each preparation was heated in a water bath at 80°C for 30 min, then centrifuged at 3,500 rpm for 15 min and stored at 4°C for future analysis.

### Mineral content analysis

The inductively coupled plasma mass spectrometry (ICP-MS) analysis was carried out according to the methodology outlined by Türkan et al. [22]. One (1 gm) of EBN was oven-dried for 16 h at 80°C. The ash was produced by heating dry EBN in an oven at 400°C. Subsequently, 0.2 gm of ash was dissolved in a 10-mm nitric acid solution (65%). After 72 h of digestion in a tightly sealed polypropylene tube at a temperature of 90°C, the solution was condensed to approximately 1 ml and diluted with ultrapure water to a final amount of 20 ml. ICP-MS analysis was performed on 25 mineral elements.

### 3-[4,5-dimethylthiazol-2-yl]-2,5-diphenyl tetrazolium bromide (MTT) assay

The American Type Culture Collection (ATCC^®^) cell lines used in this study include the HeLa cervical cancer cell line (ATCC^®^ CCL-2™), the MCF-7 breast cancer cell line (ATCC^®^ HTB22™), and the glial regular cell line SVG p12 (ATCC^®^ CRL-8621™). After every 24 h, the cells were carefully maintained through routine passage to ensure uninterrupted growth and prevent contamination. The cytotoxic procedures were conducted following the procedure outlined by Achakzai et al. [23]. A culture flask with a high cell density was chosen, and the cells were meticulously placed in a 96-well plate at a concentration of 1 × 10_5_ cells/ml. The cells were exposed to EBN extracts at 37°C for 72 h and supplemented with 5% carbon dioxide. Control cultures were treated with solvents alone, while tamoxifen was used as a positive control. Following incubation, 50 µl of MTT solution was introduced into each well at a 2 mg/ml concentration. Subsequently, the plates were incubated for 4 h. The purple formazan crystal that had developed at the bottom of the wells was dissolved by adding 200 µl of dimethyl sulfoxide (DMSO). A spectrophotometric plate reader was employed to quantify the absorbance at a wavelength of 570 nm. The quantification of the viable cells was accomplished using the equation shown below.


$$\mathrm{Cell}\;\mathrm{viability}\;\%\;=\;\frac{\mathrm{Absorbance}\;\mathrm{of}\;\mathrm{sample}}{\mathrm{Absorbance}\;\mathrm{of}\;\mathrm{control}}\;x\;100$$

### Brine shrimp lethality (BSL) assay

The toxicity profile of the EBN extracts was evaluated by a brine shrimp mortality test utilizing five distinct EBN extract doses, ranging from 200 to 1,000 µg/ml. The assay was conducted according to the methodology outlined by Hamidi et al. [24]. A quantity of 0.5 gm of brine shrimp eggs was incubated in 500 ml of artificial seawater, which contained a 3.8% solution of sodium chloride. The incubation lasted 48 h, and the hatching was carried out under ideal conditions. Subsequently, ten active nauplii were transferred using a dropper and placed in plates containing 5 ml of saltwater and 5 ml of each concentration of EBN extract. Pure DMSO was used as the positive control. The surviving nauplii were enumerated after 24 h using a magnifying glass, and the experiment was repeated thrice. The percentage mortality and the LC_50_ were calculated using Microsoft Office Excel 2016.


$$\mathrm{Percentage}\;\mathrm{mortality}\;=\;\frac{\mathrm{tota}l\;n\mathrm{auplii}–\mathrm{survived}\;\mathrm{nauplii}}{\mathrm{tota}l\;n\mathrm{auplii}}\;\times \;100$$

### 2,2’-diphenyl-2-picrylhydrazyl (DPPH) assay

The DPPH assay was conducted following the procedure outlined by Ali et al. [25], with certain modifications. EBN samples measuring 100 µl (ranging from 1,000 to 15.625 µg/ml concentrations) were combined with a 100 µl solution of DPPH (0.01 mM) and methanol. The mixtures were placed in a dark environment and kept at 25°C for 1 h. The blank was prepared by substituting the samples with methanol. The samples were prepared in three identical sets, and the absorbance of each sample was quantified using a microplate reader at a wavelength of 517 nm. The DPPH radical scavenging activity was calculated using the formula as follows.


$$\mathrm{DPPH}\;\mathrm{scavenging}\;\mathrm{activity}\;\mathrm{(\%)}\;=\;\frac{\mathrm{Acontrol}\;–\;\mathrm{Asample}}{\mathrm{Acontrol}}\;\times \;100$$

### FRAP assay

The ferric reducing antioxidant power (FRAP) test was performed according to the methodology described by Dilshad et al. [26]. The FRAP reagent’s composition includes FeCl_3_.6H_2_O, 2,4,6-tris(2-pyridyl)-s-triazine solution, and acetate buffer. Twenty microliter of the different EBN extracts were mixed with 100 µl of FRAP reagent in a 96-well plate and kept for 30 min. A standard solution of iron (III) sulfate was used as a reference, and the result was determined by referencing the calibration curve, which spanned from 0.2 to 2.0 mM. The FRAP values were reported as mM ferrous equivalents Fe (II).

### Statistical analysis

The experiments were performed in triplicate, and the results were displayed alongside the average value and its accompanying SD. The data and graph were analyzed using Microsoft Office Excel 2016 (version 16.0) and one-way analysis of variance in IBM SPSS Statistics (v21.0.0).

## Results

### Mineral analysis

**Figure 1.**
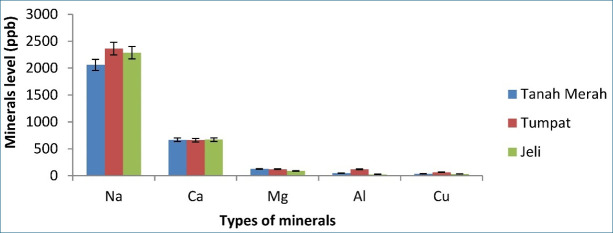
Minerals level (Na, Ca, Mg, Al, and Cu) of raw EBN samples from Tanah Merah, Tumpat, and Jeli.

**Figure 2.**
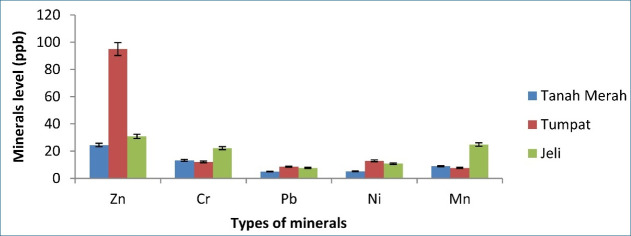
Minerals level (Zn, Cr, Pb, Ni, and Mn) of raw EBN samples from Tanah Merah, Tumpat, and Jeli.

**Figure 3.**
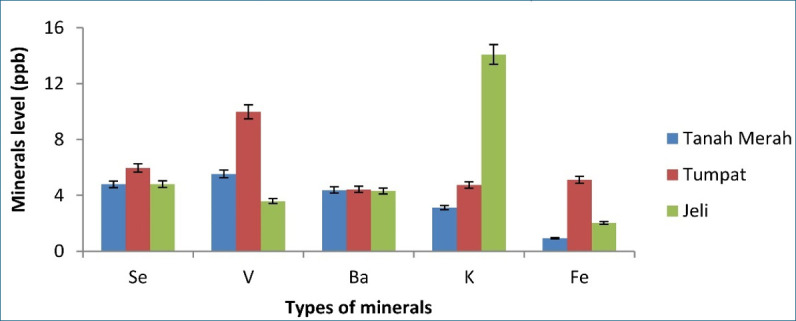
Minerals level (Se, V, Ba, K, and Fe) of raw EBN samples from Tanah Merah, Tumpat, and Jeli.

**Figure 4.**
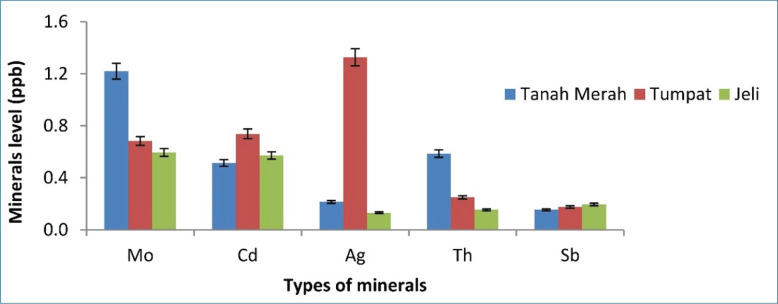
Minerals level (Mo, Cd, Ag, Th, and Sb) of raw EBN samples from Tanah Merah, Tumpat, and Jeli.

**Figure 5.**
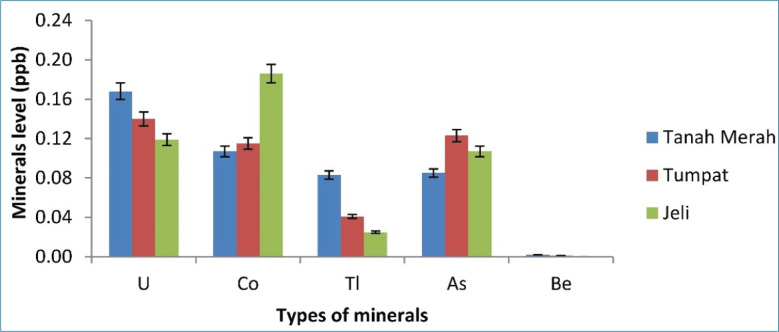
Minerals level (U, Co, Tl, As, and Be) of raw EBN samples from Tanah Merah, Tumpat, and Jeli.

Figures 1–5 depict a bar chart of 25 minerals detected in EBN extracts, including macronutrients [calcium (Ca), aluminum (Al), magnesium (Mg), sodium (Na), and potassium (K)]. Microelements [zinc (Zn), copper (Cu), iron (Fe), cobalt (Co), manganese (Mn), selenium (Se), chromium (Cr), nickel (Ni), vanadium (V), barium (Ba), molybdenum (Mo), argentum (Ag), and beryllium (Be)] were also detected. Heavy metals [cadmium (Cd), arsenic (As), thallium (Tl), lead (Pb), thorium (Th), antimony (Sb), and uranium (U)] were found in trace concentrations. From the results of this study, the TU EBN sample recorded the highest Na content (>2,000 ppb), as shown in Figure 1; it also has the highest Zn content (90 ppb) (Fig. 2). However, the JE EBN sample had the highest K content (15 ppb), as illustrated in Figure 3. Similarly, the TU EBN sample has the highest Ag content (15 ppb) (Fig. 4). The JE EBN sample showed the highest concentration of Co (0.18 ppb), as shown in Figure 5.

### MTT assay

Table 1 shows the IC_50_ values of three EBN extracts from TM, TU, and JE on cancerous (HeLa and MCF-7) and non-cancerous (SVG p12) cell lines. The cytotoxicity test showed that JE HCl extract had a significantly lower IC_50_ value of 20.00 ± 0.57 µg/ml against HeLa cell lines compared to other extracts, indicating its potential as a potent anticancer agent. Similarly, TU NaOH extract remarkably affected the MCF-7 cell lines IC_50_ (0.03 ± 0.01 µg/ml). Nevertheless, the JE aqueous extract showed the lowest efficacy against the cancer cells (MCF-7), as indicated by its IC_50_ (32.00 ± 1.00 µg/ml). The EBN extracts demonstrated no adverse effects on non-cancerous SVG p12 cell lines (>100 µg/ml), indicating its selective safety profile.

### BSL test

Table 2 presents the BSL results, indicating that the LC_50_ values for all EBN extracts from different locations exceeded 1,000 µg/ml. This finding suggests that the EBN extracts exhibit minimal toxicity and are considered safe for consumption.

### Antioxidant assay (DPPH and FRAP)

The antioxidant activity of EBN extracts was evaluated through DPPH and FRAP assays, revealing variability influenced by extraction methods and geographical origin. The DPPH assay results indicated that aqueous TM and TU extracts demonstrated the highest free radical scavenging activity, with an IC_50_ value of 10.0 µg/ml. In contrast, TM and JE HCl extracts exhibited the lowest activity, with an IC_50_ value of 40.0 µg/ml (Table 3). In the FRAP assay, NaOH extracts exhibited the highest reducing power, especially for TU (1.38 ± 0.00 mM gallic acid equivalents [GAE] gm⁻¹), while HCl extracts from all locations showed limited activity (0.07–0.08 mM GAE gm⁻¹) (Table 4).

## Discussion

The results of this study offer significant insights into the mineral composition, cytotoxic effects, and antioxidant potential of *A. fuciphagus* nests sourced from various regions in Kelantan, Malaysia. Numerous essential mineral elements were detected in the EBN extracts. Likewise, the extracts displayed potent cytotoxic effects on the cancer cell lines without affecting the normal cell lines (SVG p12). This demonstrates the medicinal potential and safety of EBN. Our study reveals that EBN exhibits antioxidant properties, as evidenced by its activity in DPPH and FRAP assays. EBN possesses many macro- and microelements, such as Na, P, Ca, K, Zn, Fe, Co, and Se. Heavy metals, including As, Pb, and Cd. Nevertheless, these heavy metals were within the acceptable limit [20]. EBN from TU showed the highest Na, Zn, and Ag content. The JE EBN sample had the highest amount of K and Mn, whereas the EBN from TM contained more Mo than the other samples. The geographical variations in mineral content observed in the study may reflect the influence of local environmental conditions, such as swiftlet dietary patterns. Lahjie et al*.* [8] proposed that swiftlet diets vary based on regional vegetation and food availability, which could explain these disparities. Sodium (Na) is crucial in facilitating nerve impulses, managing the appropriate balance of water and minerals in the body, and enabling muscular functions [27].

**Table 1.** IC_50_ values of three samples of EBN extracts on cancerous (HeLa and MCF-7) and non-cancerous (SVG p12) cell lines.

EBN sample	Cell line	IC_50_ value of EBN extracts and control (µg/ml)			
		Aqueous	NaOH	HCl	Tamoxifen
Tanah Merah	HeLa MCF-7 SVG p12	>100 3.00 ± 1.04 >100	32.00 ± 1.53 0.05 ± 0.01 >100	28.00 ± 2.08 0.60 ± 0.05 >100	0.02 ± 0.01 0.02 ± 0.03 -
Tumpat	HeLa MCF-7 SVG p12	>100 0.05 ± 0.01 >100	35.00 ± 1.53 0.03 ± 0.01 >100	60.00 ± 1.00 0.39 ± 0.01 >100	0.02 ± 0.01 0.02 ± 0.03 -
Jeli	HeLa MCF-7 SVG p12	>100 32.00 ± 1.00 >100	22.00 ± 2.52 7.00 ± 0.50 >100	20.00 ± 0.57 2.00 ± 0.06 >100	0.02 ± 0.01 0.02 ± 0.03 -

**Table 2.** Brine shrimp lethality assay (LC_50_) value of EBN extract.

EBN samples	EBN extracts LC_50_ values (µg/ml)		
	Distilled water	NaOH	HCl
Tanah Merah	>1,000	>1,000	>1,000
Tumpat	>1,000	>1,000	>1,000
Jeli	>1,000	>1,000	>1,000

K is essential in maintaining fluid equilibrium, facilitating neuronal transmission, and enabling muscular contraction. It also offers prophylactic advantages against osteoporosis, stroke, and kidney problems [28]. Zn serves as a stimulator for immune response and acts as a stabilizer for cell membranes. Moreover, Zn is an integral part of synthesizing DNA and RNA polymerase. Furthermore, both Zn and Co can be used as cofactors for enzymatic activity [27]. Our study expands upon the findings of Hun et al. [20] and Quek et al. [21], providing a more comprehensive mineral profiling of EBNs from Kelantan. While previous studies highlighted Na and Ca as dominant minerals, our research identifies regional variations, such as higher K levels in JE EBN (>2,000 ppb) and elevated Zn levels in TU EBN (90 ppb). These differences highlight the influence of geographical and environmental factors on EBN composition, a perspective not extensively explored in earlier studies. Furthermore, the high levels of these minerals detected in this study may contribute to the observed antioxidant and cytotoxic effects, potentially through mechanisms involving oxidative stress reduction and apoptosis induction in cancer cells [3,29].

Based on the cytotoxicity study results, EBN JE HCl extract significantly showed the lowest IC_50_ value of 20.00 ± 0.57 µg/ml against the HeLa cell line; meanwhile, EBN TU NaOH extract showed the lowest IC_50_ value of 0.03 ± 0.01 µg/ml against the MCF-7 cell line. The IC_50_ values for EBN extracts differ significantly between most location pairs, except for TU and JE in the NaOH extract for the HeLa cell line. This suggests that geographical origin influences the cytotoxic effectiveness of EBN extracts. According to the National Cancer Institute, an IC_50_ less than 20 µg/ml of crude extract is considered active against cancer cells [30]. Therefore, the significant cytotoxic effects of JE HCl and TU NaOH extracts, with IC_50_ values of 20.00 ± 0.57 µg/ml and 0.03 ± 0.01 µg/ml, respectively, demonstrate the potential of EBN extracts as targeted therapeutic agents for cervical and breast cancers. Notably, the absence of toxicity in non-cancerous SVG p12 cell lines highlights the EBN extracts’ selective safety profile. However, these findings of observed in vitro activity require validation through preclinical and clinical studies to determine efficacy in living systems.

According to Dai et al. [5], EBN has immuno-enhancing properties, which can potentially treat human breast cancer. Moreover, a previous study reported the presence of galactose or N-galactosamine and lectin-containing sugar chains in EBN with diverse pharmacological effects [17]. Moreover, Huang et al. [31] reported that impurities incorporated into EBN and the different nutrition contents between regions and nest types may affect cell proliferation. The BSL assay results demonstrated that all the EBN extracts were non-toxic and deemed safe for consumption, as their LC_50_ values exceeded 1,000 µg/ml. Clarkson defined the toxicity as non-toxic if the LC_50_ is higher than 1,000 µg/ml, mild toxicity if the LC_50_ is between 500 and 1,000 µg/ml, highly toxic if the LC_50_ is between 100 and 500 µg/ml, and extremely toxic if the LC_50_ is between 0 and 100 µg/ml [32]. The IC_50_ value for antioxidant activity is the sample concentration that can inhibit 50% of DPPH scavenging activity. The lowest value of IC_50_ indicates the highest antioxidant capacity. Therefore, the TU and TM EBN aqueous extracts (IC_50_ 10 µg/ml) could be a potential candidate for nutraceutical development due to their low IC_50_ values. The EBN aqueous extracts could be used as dietary supplements to manage oxidative stress-related conditions. These applications have the potential to meet the increasing demand for natural bioactive compounds in the global nutraceutical and pharmaceutical markets.

**Table 3.** Antioxidant activity of EBN extracts determined by DPPH Assay.

Extracts	Tanah Merah	Tumpat	Jeli	Gallic acid (control)
IC_50_ (µg/ml)				
Aqueous	10.0 ± 0.02	10.0 ± 0.01	30.0 ± 0.00	
NaOH	20.0 ± 0.02	20.0 ± 0.00	20.0 ± 0.01	< 10.0 ± 0.00
HCl	40.0 ± 0.09	10.0 ± 0.04	40.0 ± 0.00	

The values are the mean ± SD from three replicates (*n = *3).

**Table 4.** Antioxidant activity of EBN sample extracts determined by FRAP Assay.

Extracts	Tanah Merah	Tumpat	Jeli	Gallic acid
(mM GAE gm^-1^)				
Distilled water	0.20 ± 0.02	0.15 ± 0.03	0.20 ± 0.01	
NaOH	1.35 ± 0.04	1.38 ± 0.00	1.23 ± 0.00	1.96 ± 0.02
HCl	0.08 ± 0.09	0.07 ± 0.02	0.07 ± 0.00	

The values are presented as mean ± SD from three replicates (*n =* 3).

Our results agree with a report by Hun et al. [20], who documented the antioxidant properties of EBN extracts at concentrations spanning from 0.059 to 1.0981 mM/l. Furthermore, previous research documented the antioxidant properties of EBN from Peninsular Malaysia, with DPPH scavenging activities reported between 2.33 and 3.49 mg AAE/gm and FRAP values of 6.17 and 10.37 mg AAE/gm [21]. Comparatively, our study demonstrates significantly strong antioxidant efficacy, with DPPH IC_50_ values as low as 10 µg/ml for aqueous extracts and a ferric reducing power of 1.38 ± 0.00 mM GAE gm⁻¹ in NaOH extract from TU. These results suggest that EBN from these districts may possess unique bioactive compounds that enhance their antioxidant activity. It was reported that EBN possesses essential proteins with long chains of amino acids that have significant antioxidant activity [33]. The presence of the amino acids phenylalanine, tryptophan, proline, and histidine in EBN has been found to have a considerable positive relationship with antioxidant activity [25]. These findings are particularly noteworthy, as previous studies mainly concentrated on EBN’s nutritional and antioxidant potential, with limited exploration of its potential as a natural anticancer agent.

## Conclusion

The predominant mineral elements detected in EBN extracts were Na, Ca, and K. The EBN samples from JE and TU exhibited potent inhibition of cancerous cell lines (HeLa and MCF-7) compared to the EBN sourced from TM. However, the TM and TU EBN samples exhibited better free radical scavenging activity than those from JE in the DPPH assay. However, the NaOH extract from TU had a higher ferric reducing antioxidant power than the other EBN extracts. All the EBN samples were nontoxic and safe for consumption. Thus, EBN may be considered an alternative anticancer and antioxidant agent. Although our findings offer significant insights into the bioactivity of EBN extracts, future research should incorporate a wider sampling range and explore long-term safety and efficacy of EBNs in animal models and human clinical trials.

## List of abbreviations

ATCC, American Type Culture Collection; BSLA, brine shrimp lethality assay; DMSO, dimethyl sulfoxide; DPPH, 2,2’-diphenyl-2-picrylhydrazyl; EBN, edible bird’s nest; FRAP, ferric-reducing antioxidant power; GAE, gallic acid equivalents; HCl, hydrochloric acid; IC_50_, half-maximal inhibitory concentration; ICP-MS, inductively coupled plasma mass spectrometry; JE, Jeli; LC_50_, lethal concentration 50%; MTT, 3-[4,5-dimethylthiazol-2-yl]-2,5 diphenyl tetrazolium bromide; NaOH, sodium hydroxide; TM, Tanah Merah; TU, Tumpat.
